# Pretreatment with platelet-rich plasma protects against ischemia–reperfusion induced flap injury by deactivating the JAK/STAT pathway in mice

**DOI:** 10.1186/s10020-024-00781-3

**Published:** 2024-02-01

**Authors:** Linlin Su, Songtao Xie, Ting Li, Yanhui Jia, Yunchuan Wang

**Affiliations:** Department of Burns and Cutaneous Surgery, Xijing Hospital, Air Force Medical University, No.127 Changle West Road, Xincheng District, Xi’an, 710032 Shaanxi China

**Keywords:** Platelet-rich plasma, Ischemia–reperfusion, Apoptosis, Inflammatory response, Oxidative stress

## Abstract

**Background:**

Ischemia–reperfusion (I/R) injury is a major cause of surgical skin flap compromise and organ dysfunction. Platelet-rich plasma (PRP) is an autologous product rich in growth factors, with tissue regenerative potential. PRP has shown promise in multiple I/R-induced tissue injuries, but its effects on skin flap injury remain unexplored.

**Methods:**

We evaluated the effects of PRP on I/R-injured skin flaps, optimal timing of PRP administration, and the involved mechanisms.

**Results:**

PRP protected against I/R-induced skin flap injury by improving flap survival, promoting blood perfusion and angiogenesis, suppressing oxidative stress and inflammatory response, and reducing apoptosis, at least partly via deactivating Janus kinase (JAK)-signal transducers and activators of transcription (STAT) signalling pathway. PRP given before ischemia displayed overall advantages over that given before reperfusion or during reperfusion. In addition, PRP pretreatment had a stronger ability to reverse I/R-induced JAK/STAT activation and apoptosis than AG490, a specific inhibitor of JAK/STAT signalling.

**Conclusions:**

This study firstly demonstrates the protective role of PRP against I/R-injured skin flaps through negative regulation of JAK/STAT activation, with PRP pretreatment showing optimal therapeutic effects.

## Introduction

Skin flaps are commonly used in reconstructive surgery to close defects, but partial or complete necrosis is a common problem (Robertson et al. 2017). Axial flaps with confidential pedicles are more vulnerable to ischemia–reperfusion (I/R) injury (Tatlidede et al. 2009), which is caused by inadequate blood perfusion, oxidation stress, inflammation, and apoptosis (Lee et al. 2022). Management of necrotic flaps is time-consuming and requires repetitive dressings and surgery (Finkemeier and Neiman 2016). Therefore, mitigating I/R-induced flap injury is a clinical challenge, also seen in organ transplantation.

Platelet-rich plasma (PRP) is a source of various growth factors that can be locally released for up to three weeks (Etulain 2018). Autologous PRP is biocompatible and safe without causing immunoreaction or infection (Etulain 2018; Everts et al. 2020). Many drugs and growth factors tested in animal models have limited clinical use because of high cost and short half-life. Many clinical devices are currently available to automatically collect and process PRP at low cost (Gomri et al. 2022; Magalon et al. 2021). PRP has been used in clinic for years to improve wound healing, bone regeneration, oxidative stress, inflammation, and blood loss (He et al. 2022; Anitua et al. 2022). Therefore, PRP could represent a novel and effective therapy for managing I/R-induced flap injury.

PRP has been showed to reduce pancreatic biochemical and histopathological alterations caused by renal I/R injury and could be a promising therapy for pancreatic damage (Shehata et al. 2022). PRP effectively minimizes ovarian damage and preserves ovarian reserves following ovarian torsion in I/R-induced ovary injury in rats (Bostancı et al. 2022). PRP also protects testicular tissues against torsion-resulted I/R by inhibiting neutrophil infiltration and oxidative stress and increasing antioxidant defense (Sekerci et al. 2017). Studies also indicate the beneficial roles of PRP in reducing I/R injuries in the heart (Hargrave et al. 2016) and liver (Aydın et al. 2019). However, there have been few studies on the protective role of PRP and the optimal timing for its administration in I/R-injured skin flap models.

Various growth factors are released and actively involved in the process of skin injury and repair. Epidermal growth factor (EGF) plays a crucial role in dermal wound repair by stimulating keratinocyte proliferation and migration, and accelerating wound healing when used in partial or full-thickness skin wounds (Sezgin et al. 2017). Vascular endothelial growth factor (VEGF) improves the survival rate of skin flap and salvages greater peripheral segment of the flap by inducing neovascularization (Vourtsis et al. 2012). Transforming growth factor (TGF)-β1 contributes to epidermal and dermal thickening and cellular turnover during cutaneous wound healing (Chong et al. 2020). Platelet-derived growth factor (PDGF) is a major proliferative and migratory stimulus for connective tissue cells during the initiation of skin repair processes (Rollman et al. 2003). Insulin-like growth factor (IGF)-1 is beneficial for wound re-epithelialization, granulation tissue formation, collagen deposition, and angiogenesis (Roberts et al. 2021). It is reasonable to assume that the application of PRP can promote skin flap survival by releasing these growth factors. The Janus kinase (JAK)-signal transducers and activators of transcription (STAT) pathway is a stress-responsive mechanism that transduces signals from the cell surface to the nucleus, modulating gene expression. It has been implicated in various I/R injuries (Bolli et al. 2003; Li et al. 2022; Xiong et al. 2023; Domingos et al. 2023). Therefore, the current study aimed to investigate the effect of PRP on I/R injury in a mouse axial flap model, determine the optimal timing for PRP administration, and explore its potential mechanism.

## Materials and methods

### Animals

Fifty-six healthy male C57BL/6N mice, weighing 25 ~ 30 g at 8-week-old, were purchased from the Experimental Animal Center of the Air Force Medical University (Xi' an, China). The mice were housed under standard laboratory conditions (temperature: 25 °C ± 2 °C, relative humidity: 55%, 12-h light/12-h dark cycle) with free access to food and water, and acclimated for one week before the experiment. Animal experiments were conducted in accordance with the Ethics Committee of Xijing Hospital affiliated to the Air Force Medical University (Approval no. KY20203237-1) and the National Institutes of Health Guide for the Care and Use of Laboratory Animals (2011). The mice were euthanized by CO_2_ asphyxiation at a flow rate of 30% chamber vol/min. The humane endpoints include a reduction of 4 ~ 6 °C in body temperature, a weight loss of > 10%, decreased activity (lethargy) and alertness, a rough coat and hunched posture.

### PRP preparation

Mice were anesthetized with 2.5% isoflurane. PRP was prepared from the whole bloods that were drawn from the retro-orbital plexus of mice into ethylene diamine tetraacetic acid (EDTA)-coated Eppendorf (EP) tubes, mixed by inversion, and centrifuged at 160 × *g* for 10 min at room temperature to separate plasma (superior layer) from red blood cells (inferior layer) and white blood cells (intermediate layer). Next, the plasma plus platelets were collected using a sterile syringe, transferred to a new EP tube without anti-coagulant, and centrifuged again at 400 × *g* for 10 min at room temperature, yielding PRP with a mean concentration of 975, 000 platelets/μl measured by an automated hematology analyzer (BC-6000Plus; Mindray, Shenzhen, China). Activated PRP was prepared by adding 500 μL of 10% CaCl_2_ solution to 20 mL of PRP solution. This PRP contains soluble fibrin, fibronectin, and vitronectin, forming a biologically active matrix conducive to tissue repair (Leo et al. 2015).

### Surgical procedure, grouping and drug treatment

On the day of the operation (designated as day 0), mice were anesthetized with 30 mg/kg of pentobarbital sodium via intraperitoneal (*i.p.*) injection following initial anesthesia with isoflurane. They were then placed on a heating pad to maintain a constant body temperature throughout the surgery. After thoroughly cleaning the entire dorsal area, a lateral thoracic artery-based pedicled skin flap measuring 2.5 cm × 4 cm was raised in the caudal to cranial direction by careful dissection with direct visualization of pedicle. This axial flap was comprised of skin, subcutaneous tissue, and panniculus carnosus muscle. After flap elevation, a medical-grade silicon sheet was placed over the muscle bed as a barrier. In the non-I/R groups, flaps were subcutaneously injected with a single dose of 150 μl of phosphate buffer saline (PBS, vehicle control) or PRP (designated as 0 h on day 0), and sutured into the original place using 4–0 polyglactin sutures. In I/R groups, after flap elevation, the pedicle was clamped with a microclamp for 4 h to induce ischemia. The clamp was then removed to allow the reperfusion for 12 h. The flaps were then sutured back to the original position. In I/R groups, 150 μl of PBS or PRP was subcutaneously injected into the flaps at three different time points: prior to the ischemia (0 h on day 0), immediately before reperfusion (4 h on day 0), or during reperfusion (10 h on day 0). Thirty-two mice were randomly divided into eight groups with n = 4 in each group: Group 1 received PBS without I/R; Group 2 received PRP without I/R; Group 3 received PBS pretreatment with I/R; Group 4 received PRP pretreatment with I/R; Group 5 received PBS mid-treatment with I/R; Group 6 received PRP mid-treatment with I/R; Group 7 received PBS post-treatment with I/R; Group 8 received PRP post-treatment with I/R.

In experiments involving AG490, AG490 (HY-12000; MedChemExpress, Monmouth Junction, NJ, USA) was dissolved in dimethyl sulfoxide (DMSO) and further diluted in PBS to achieve a final working concentration of 10 mg/ml. Flaps were injected with AG490 solution (5 mg/kg b.w.) subcutaneously with a final volume of 150 μl (Zhang et al. 2023). Twenty-four mice were randomly divided into six groups with n = 4 in each: Group 1 received PBS without I/R; Group 2 received PRP without I/R; Group 3 received AG490 without I/R; Group 4 received PBS pretreatment with I/R; Group 5 received PRP pretreatment with I/R; Group 6 received AG490 pretreatment with I/R.

### Measurement of oxidant/anti-oxidant parameters

*Malondialdehyde (MDA):* MDA is an end product of lipid peroxidation and has been used as an oxidative stress marker. Here, the MDA content in the skin flap was measured to quantify the change in oxidative stress damage after PRP treatment. Tissue samples were suspended in Tris–HCl buffer at a ratio of 1:5 (w/v) and minced with surgical scissors for 15 s on an ice-cold plate. The resulting suspensions were homogenized for 2 min at 4 °C with an automatic homogenizer and centrifuged for 15 min at 12,000 rpm. The supernatants were then collected to determine MDA content using a MDA colorimetric assay kit (TBA method; Nanjing Jiancheng Bioengineering Institute, Nanjing, China). The absorbance of each sample was measured at 586 nm. Results were presented as nanomoles of MDA per gram of tissue (nmol/g).

*Nitric oxide (NO):* NO content was measured using a Nitric Oxide colorimetric assay kit (BioVision, Mountain View, CA, USA), which provides a convenient assessment of total nitrate/nitrite in two steps. In the first step, nitrate was converted to nitrite using nitrate reductase. In the second step, Griess Reagents were used to convert nitrite to a deep purple azo compound. The amount of the azo chromophore accurately reflected the NO amount in samples. The resulting azo dye had a bright reddish-purple color which could be measured at 540 nm. Results were presented as micromoles of NO per gram of tissue (μmol/g).

*Superoxide dismutase (SOD) and glutathione peroxidase (GSH-Px) activities:* Each tissue sample (0.5 g) was homogenized at 4 °C. Then the homogenates were centrifuged for 15 min at 12,000 rpm, and the supernatants were harvested and kept frozen at -80 °C, pending measurement of SOD and GSH-Px activities.

SOD activity was detected according to the instructions of a commercial kit (Nanjing Jiancheng Bioengineering Institute). This method utilizes xanthine and xanthine oxidase to generate superoxide radicals, which react with INT (idophenyl–nitrophenol–phenyltertrazolium) to form a red formazan dye. SOD activity was assessed by the degree of inhibition of this reaction, which was measured by the absorbance at 505 nm at 37 °C. Results were presented as units of SOD activity per gram of tissue (U/g).

GSH-Px activity was determined using a spectrophotometer as described in the kit (Nanjing Jiancheng Bioengineering Institute). This enzyme catalyzes the oxidation of glutathione (GSH) by hydrogen peroxide. The presence of glutathione reductase (GSR) and NADPH facilitates the conversion of oxidized glutathione (GSSG) to its reduced form. The absorbance was measured at 340 nm. The results were expressed as units of GSH-Px activity per gram of tissue (U/g).

### Enzyme-linked immunosorbent assay (ELISA)

ELISA kit for VEGF was commercially purchased from Beyotime Biotechnology (Shanghai, China) and used to determine its tissue content. A 100-µl aliquot of the supernatant from each sample was collected for measurement. The concentration of VEGF in the supernatant was normalized to the protein concentration in the respective sample. The absorbance was measured at 450 nm using a Model 680 microplate reader (Bio-Rad Laboratories, Inc., Hercules, CA, USA).

### Hematoxylin and eosin (H&E) staining

The flap tissues were collected from the surviving regions of the flaps. The samples were fixed with 4% paraformaldehyde in 0.1 mol/L phosphate buffer for 24 h, embedded in paraffin wax, cut into 5-µm sections with a microtome, and mounted onto poly L-lysine-coated slides. Then, the skin flap sections were dewaxed with xylene and treated with ethanol at different concentrations for 5 min. Next, these sections received hematoxylin staining for 5 min, 5% acetic acid treatment for 1 min, eosin staining for 1 min, and rinsed with running water. Finally, the sections were dehydrated in 70%, 80%, 90%, 100% ethanol for 1 min each, and xylene for 1 min. Images were captured under a light microscope (Olympus Co., Tokyo, Japan).

### TUNEL staining

The skin flap samples were embedded in paraffin and sectioned at a thickness of 5 μm, as previously described. A TUNEL labeling kit was commercially purchased from Roche (Penzberg, Germany) and used to detect cellular apoptosis according to the manufacturer’s instructions. In brief, the sections were incubated with a reaction mixture containing terminal deoxynucleotidyl transferase (TdT) and FITC-conjugated deoxyuridine triphosphate (dUTP) for 1 h at 37 °C. The nuclei were stained with DAPI before the slides were mounted. TUNEL-positive cells were then observed under an inverted fluorescence microscope (Olympus IX71; Olympus Co.). For quantification analysis, images were processed with Image-Pro Plus software (Media Cybernetics, Silver Spring, MD, USA). Fields of view were randomly selected by an observer blinded to the experimental groups, and the TUNEL-positive cells and total cells were counted. The TUNEL-positive cell percentage was calculated as follows: (the number of TUNEL-positive cells/ the total cell number) × 100%.

### Immunohistochemistry (IHC)

The flap sections at a thickness of 5 μm were deparaffinized in xylene and then rehydrated in a graded ethanol series. After washing, 3% hydrogen peroxide solution was added to the sections to block endogenous peroxidase activity. Then, sodium citrate buffer (pH 6.0) was used for antigen retrieval by heating at 95 °C for 20 min. After blocking with 10% (w/v) bovine serum albumin in PBS for 10 min, the sections were incubated with mouse anti-hypoxia-inducible factor (HIF)-1α primary antibody at an appropriate dilution at 4 °C overnight. The next day, the slides were further incubated with horseradish peroxidase (HRP)-conjugated second antibody at room temperature for 1 h, stained with a DAB detection kit, and then counterstained with hematoxylin. Images were captured using a microscope at 200 × magnification (Olympus Co.).

### RNA isolation and quantitative real-time polymerase chain reaction (qRT-PCR)

Flap samples were harvested for each group for the analysis of inflammatory cytokine expression. Total RNAs (5 μg) were extracted from each sample using Trizol reagent (Invitrogen, Waltham, MA, USA) and reverse transcribed into cDNA in a 20-μl reaction mixture using an RT reagent kit (Takara, Shiga, Japan). qRT-PCR was performed in a Real-Time PCR machine (iQ5; Bio-Rad Laboratories, Hercules, CA, USA) using a SYBR green PCR mastermix (Takara Bio Inc, Shiga, Japan). The specificity of PCR products was verified by melting curve analysis. As an internal control, the levels of 18S were quantified in parallel with the target genes. Normalization and fold changes were calculated using the comparative Ct method. Each sample was analyzed in triplicate. Primer pairs were designed and listed in Table 1.

**Table 1:** Primer sequences for qRT-PCR in this study

Gene	Forward primer	Reverse primer
*TNF-a*	5ʹ- TTGTGTTAGCTTAGCCCGATCGTA -3ʹ	5ʹ- CCGTCGTAAAAGCTAGTCGATC -3ʹ
*IL-6*	5ʹ- ACGTAGCTAGCTAGTCGGTATG -3ʹ	5ʹ- TCGTAGCTTGGCTAGTCGATCG -3ʹ
*MCP-1*	5ʹ- ACTAGTCGATAGCTAGTCGAGCA -3ʹ	5ʹ- CCGATGCTACTAGCTAGCTAGC -3ʹ
*IL-10*	5ʹ- GTCATCGATTTCTCCCCTGTG -3ʹ	5ʹ- CCTTGTAGACACCTTGGTCTTGG -3ʹ
*GAPDH*	5ʹ- AACTTTGGCATTGTGGAAGG -3ʹ	5ʹ- GGATGCAGGGATGATGTTCT -3ʹ

### Western blot analysis

Flap samples were harvested from each treatment group and lysed in IP lysis buffer supplemented with protease and phosphatase inhibitor cocktails. The tissue lystes were then centrifuged, and the supernatants were collected. The protein concentration was determined using a Model 680 microplate reader (Bio-Rad). Then, 40 μg of proteins in each sample were separated on a 10% SDS–polyacrylamide gel (PAGE) and electrotransferred to polyvinylidene difluoride (PVDF) membranes. After blocking in 5% non-fat milk in TBST for 1 h, the membranes were incubated with the target primary antibodies (Table 2) overnight at 4 °C. The next day, the membranes were washed three times and incubated with HRP-conjugated goat anti-rabbit or anti-mouse secondary antibodies for 1 h at room temperature. Anti-β-actin antibody was used to label the endogenous reference. The immunoreactive bands were detected and visualized by ECL reagent (Millipore, Burlington, MA, USA) and a FluorChem FC system (Alpha Innotech, Santa Clara, CA, USA). The relative band densities were measured using Image J software.

**Table 2:** Summary of primary antibodies used in this study

Gene	Catalog no.	Host	Vendor	Dilution
Bax	2772	Rabbit	Cell Signaling Technology	1:1000 for WB
Bcl-2	15071	Mouse	Cell Signaling Technology	1:1000 for WB
Bcl-xL	2764	Rabbit	Cell Signaling Technology	1:1000 for WB
HIF-1α	sc-13515	Mouse	Santa Cruz Biotechnology	1:50 for IHC
*p*-JAK2	ab32101	Rabbit	Abcam	1:1000 for WB
JAK2	ab108596	Rabbit	Abcam	1:1000 for WB
*p*-STAT1	ab109461	Rabbit	Abcam	1:1000 for WB
STAT1	ab155933	Mouse	Abcam	1:1000 for WB
*p*-STAT3	ab76315	Rabbit	Abcam	1:1000 for WB
STAT3	ab68153	Rabbit	Abcam	1:1000 for WB
β-Actin	AF0003	Mouse	Beyotime Biotechnology	1:500 for WB

### Laser Doppler flowmetry (LDF)

Laser Doppler blood flow measurement can provide deeper penetration and enable clear visualization of small vessels beneath the tissue surface, making it well-suited for angiogenesis assessment. Therefore, we employed a PeriScan PIM3 laser Doppler system (Perimed AB, Jarfalla, Sweden) to scan the entire dorsal skin area of the mice, including the flap region, and assess skin flap perfusion. The parameters of the instrument were as follows: 633 nm laser wavelength, 55 cm scan length, and 5-min scan duration. The mice were anesthetized and secured on an operating table at a temperature of 22–25 °C to expose the entire dorsal skin flap. Then, capillary blood flow in the dermis layer of mouse skin was measured using Doppler. The average blood flow was expressed in perfusion units (PU).

### Caspase-3 activity assay

Caspase-3 activity was measured using a Colorimetric assay kit (Beyotime Biotechnology). This method is based on the cleavage of the labeled caspase-3 substrate DEVD-*p*NA to form the chromophore *p*-nitroaniline (*p*NA). In brief, 40 mg of skin flap tissues were homogenized in 2 × reaction buffer and incubated for 60 min at 37 °C with the DEVD-*p*NA substrate. The amount of *p*NA produced was quantified using a spectrophotometer. The absorbance change at 400 nm was then measured and caspase-3 activity was expressed as the fold change in absorbance in the treatment group compared to the corresponding control group. Before calculating the fold change in absorbance, all background absorbance values were subtracted from the experimental results.

### Statistical analysis

All data were presented as mean ± standard deviation. The differences between two groups or among multiple groups were compared using Student’s *t*-test, or one-way analysis of variance (ANOVA) followed by Tukey–Kramer Post hoc test, respectively. All statistical analyses were performed using the SPSS 19.0 program (SPSS Inc., Chicago, IL, USA). Differences were considered statistically significant at *p* values less than 0.05.

## Results

### The establishment of skin flap I/R injury model in mice

**Fig. 1:**
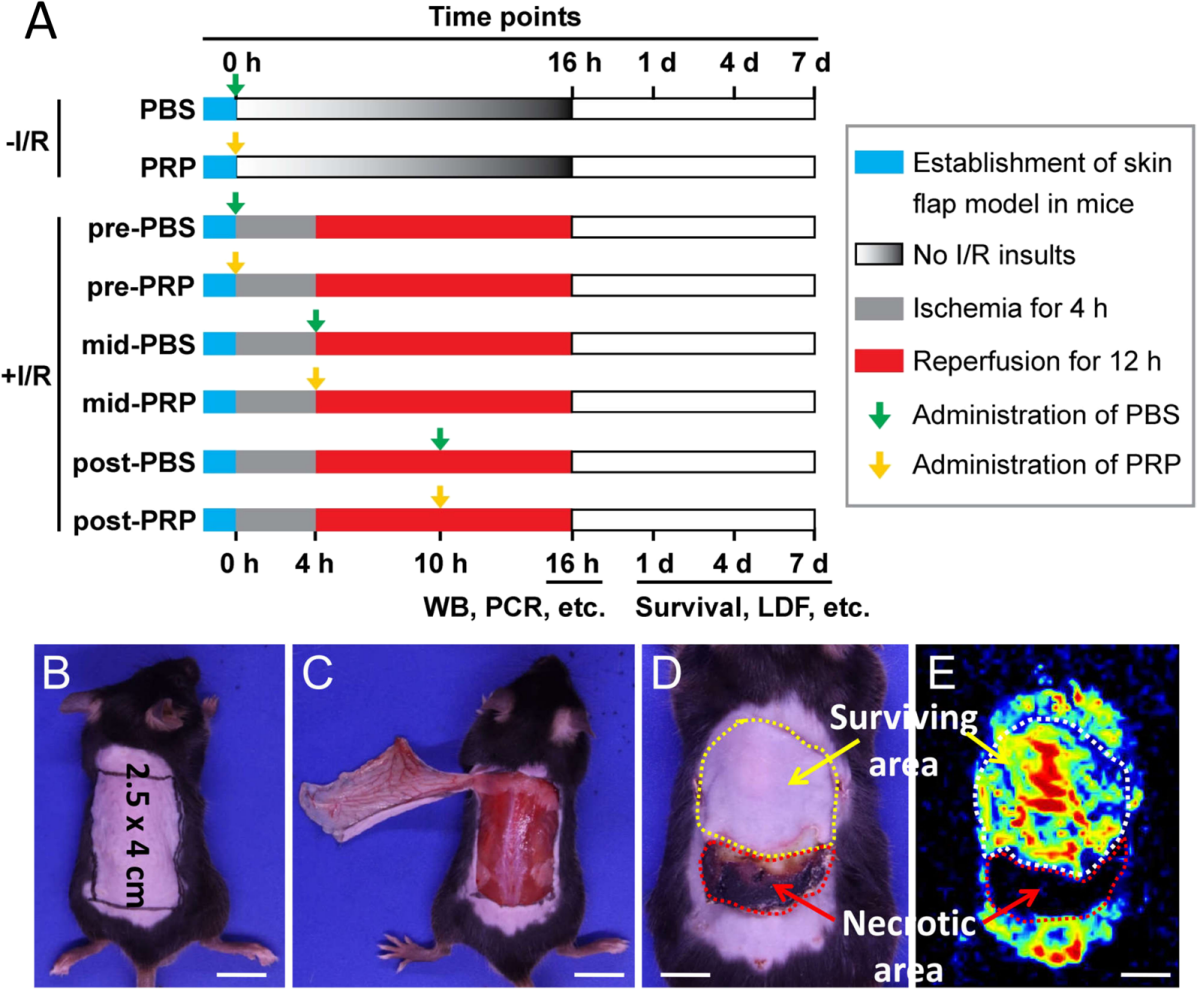
Schematic illustration of the experimental protocol and establishment of the mouse skin flap I/R model. **A** Experimental protocol: thirty-two mice were randomly and evenly divided into eight groups as described in the “Materials and methods” section. PBS (green arrows) or PRP (yellow arrows) was subcutaneously injected to the flaps at different time points with or without I/R insult. Skin flap samples collected at 16 h on day 0 were used for WB, qRT-PCR, H&E, TUNEL, and biochemical assays; flap samples collected on day 1, 4, and 7 were used to evaluate flap survival, blood perfusion, ELISA, and IHC. Blue rectangles represent the period for model establishment. Gray rectangles represent the period of 4-h ischemia. Red rectangles represent the period of 12-h reperfusion. Gradient gray rectangles represent no I/R insult. **B** Design of the lateral thoracic artery-based axial flap measuring 2.5 cm × 4 cm. **C** The flap was raised with preservation of the left lateral thoracic artery. The pedicle was exposed at the undersurface of the flap. **D** I/R-induced necrosis in the distal part of the skin flap. **E** Laser Doppler flowmetry showed blood reperfusion of the flap. Yellow and white dotted lines encircled the surviving areas. Red dotted lines encircled the necrotic areas. Scar bar = 10 mm in (**B**) and (**C**); scar bar = 6 mm in (**D**) and (**E**)

Firstly, we successfully established a skin flap model in mice, with or without I/R insult. Then we injected PRP or an equal volume of PBS subcutaneously into the flaps at different phases: prior to I/R (pre-PRP or pre-PBS treatment), immediately before reperfusion (mid-PRP or mid-PBS treatment), or during reperfusion (post-PRP or post-PBS treatment) (Fig. 1A). The model consisted of a lateral thoracic artery-based axial flap measuring 2.5 cm × 4 cm (Fig. 1B) and preservation of the left lateral thoracic artery (Fig. 1C). Following I/R insult, necrotic flap tissues were non-elastic and black or brown in color, while surviving flap portions were elastic and pink (Fig. 1D). Surviving flaps exhibited good blood circulation, while necrotic areas lacked microcirculation (Fig. 1E).

### PRP promotes the survival and blood perfusion of I/R-injured skin flaps with the highest effect when administrated prior to I/R

**Fig. 2:**
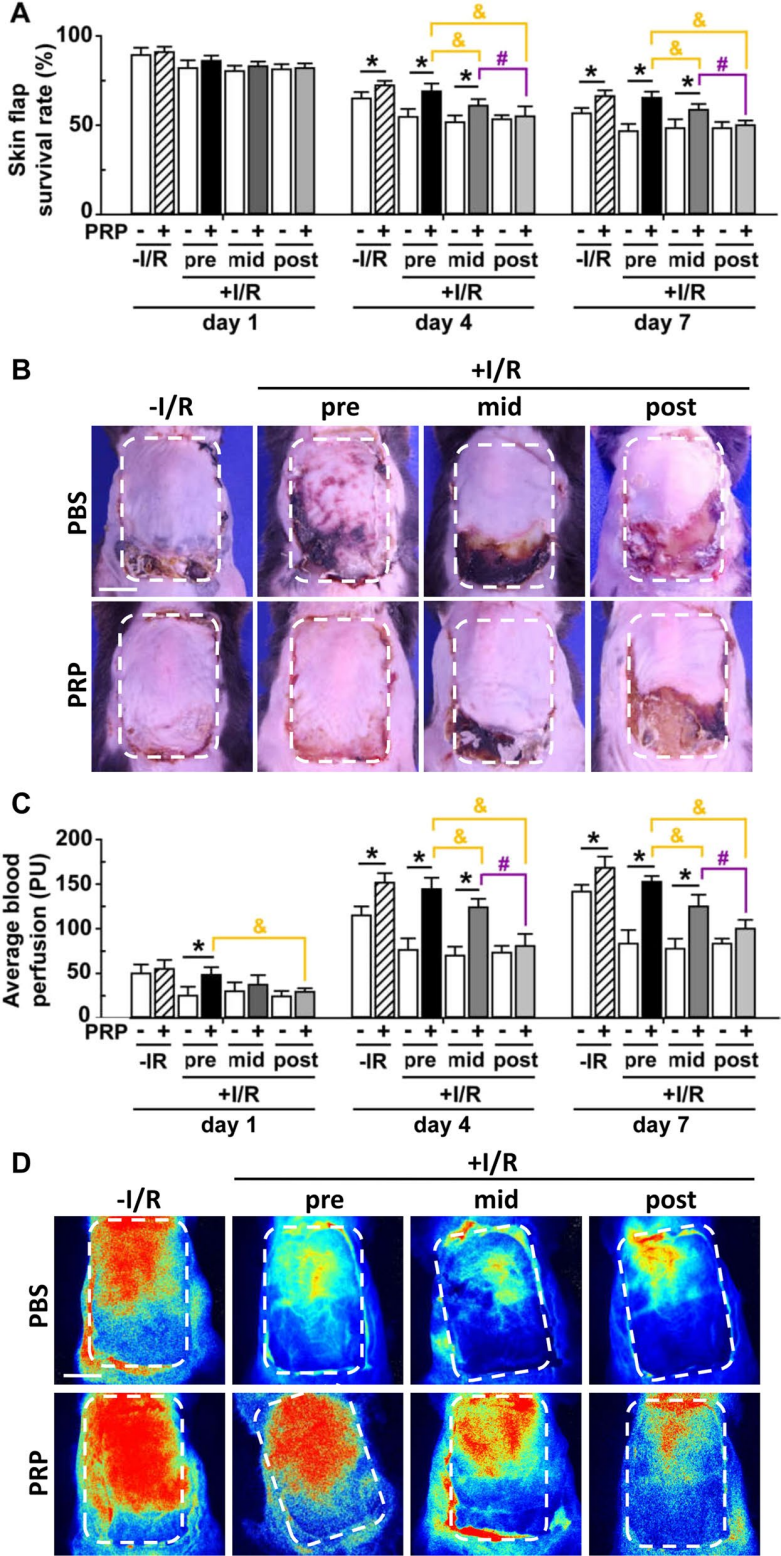
Measurement of skin flap survival rate and blood perfusion in mice treated with PRP with or without I/R insult on day 1, 4 and 7. **A** Skin flap survival rate = (survival area/total flap area) × 100%. **B** Representative images showing the flap survival on day 7. Scar bar = 1 cm. *White* dotted lines encircled the original skin flap areas. **C** Average blood perfusion of the total flap was measured using perfusion units (PUs). **D** Representative images showing the blood perfusion on day 7. Scar bar = 1 cm. *White* dotted lines encircled the original skin flap areas. Error bars represent the means ± SD (n = 4). Student’s *t*-test for comparison between two groups, and one-way ANOVA followed by the Tukey–Kramer post hoc test for comparison among multiple groups were used. *p < 0.05 vs. the corresponding PBS control, ^&^*p* < 0.05 vs. the value in pre-PRP group after I/R insult, ^#^*p* < 0.05 vs. the value in mid-PRP group after I/R insult. In each bargraph, the white bars from left to right represented treatment with PBS without I/R insult, before I/R insult, before reperfusion, or after reperfusion, respectively; the slash bar represented treatment with PRP without I/R insult; the black bar represented treatment with PRP before I/R insult; the dark grey bar represented treatment with PRP before reperfusion; the light grey bar represented treatment with PRP after reperfusion

Compared to the corresponding PBS control, PRP significantly facilitated the survival of I/R-injured skin flaps on day 4 and day 7, except in post-PRP treatment groups (Fig. 2A). PRP administrated prior to I/R had the best effects on flap survival compared to PRP administrated before reperfusion or during reperfusion (Fig. 2A). The flap survival rate on day 1 did not show any differences among all groups (Fig. 2A). Representative skin flap images on day 7 were shown in Fig. 2B. In addition, PRP significantly enhanced the average blood perfusion of I/R-injured flaps compared to the corresponding PBS control from as early as day 1 to day 7 except in the post-PRP treatment groups (Fig. 2C). PRP pretreatment had the best effects on blood perfusion compared to PRP mid-treatment or post-treatment (Fig. 2C). Representative blood perfusion images on day 7 were shown in Fig. 2D. It was worth noting that PRP also promoted the flap survival and blood perfusion in non-I/R groups on day 4 and day 7, but not on day 1 (Fig. 2A–D).

### PRP promotes angiogenesis of I/R-injured skin flaps with the highest effect when administrated prior to I/R

**Fig. 3:**
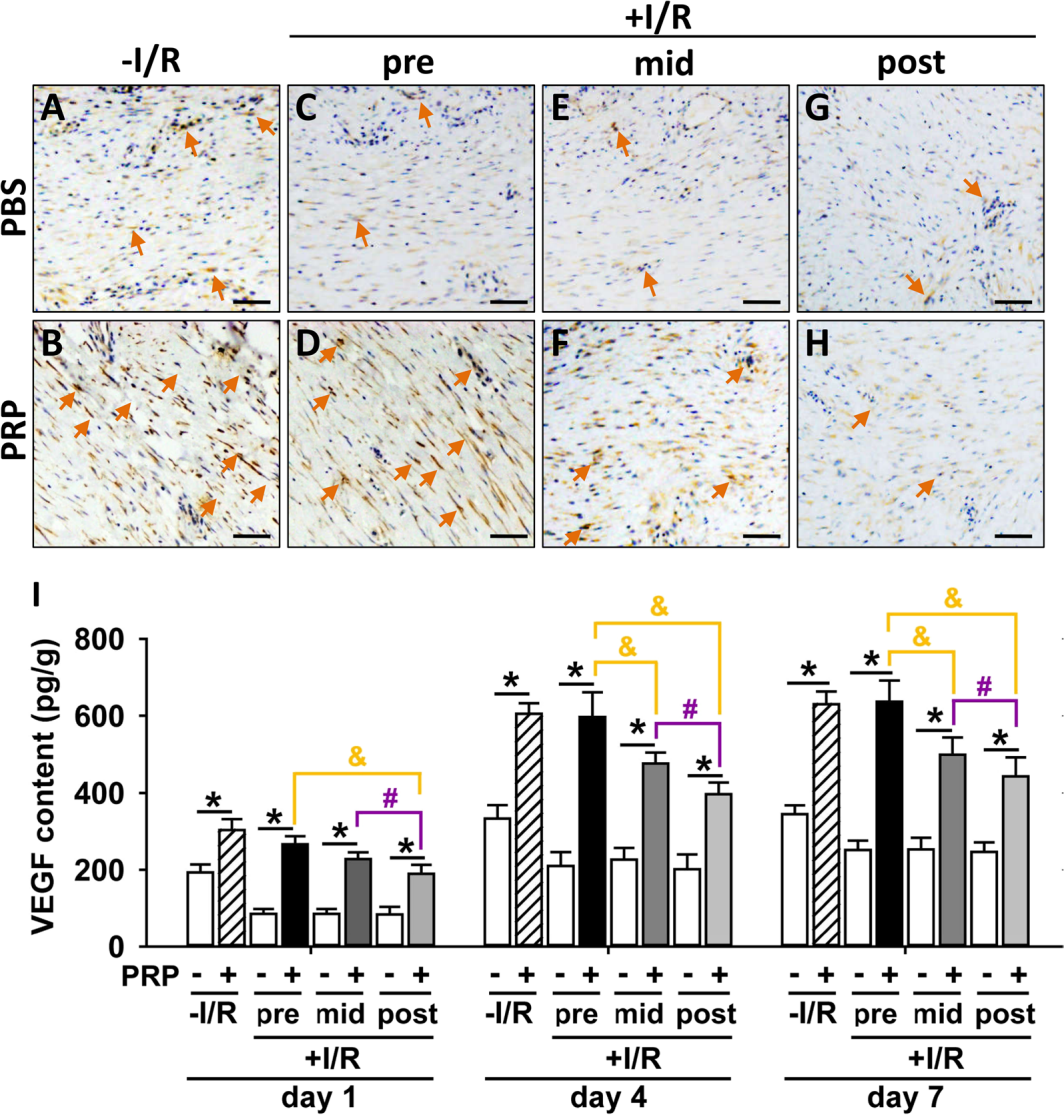
Assessment of immunohistochemical localization/expression of HIF-1α and serum content of VEGF after PRP treatment in I/R-injured mice. **A–H** HIF-1α immunohistochemical staining in different treatment groups on day 4 post-I/R. Immuno-precipitates (yellow arrows) indicate the positive HIF-1α expression, and the shade of colour represents the expression level of HIF-1α. Scar bar = 20 μm. (I) The serum levels of VEGF in different treatment groups on day 1, 4 and 7 post-I/R were assessed by ELISA. Error bars represent the means ± SD (n = 4). Student’s *t*-test for comparison between two groups, and one-way ANOVA followed by the Tukey–Kramer post hoc test for comparison among multiple groups were used. *p < 0.05 vs. the corresponding PBS control, ^&^*p* < 0.05 vs. the value in pre-PRP group after I/R insult, ^#^*p* < 0.05 vs. the value in mid-PRP group after I/R insult. In each bargraph, the white bars from left to right represented treatment with PBS without I/R insult, before I/R insult, before reperfusion, or after reperfusion, respectively; the slash bar represented treatment with PRP without I/R insult; the black bar represented treatment with PRP before I/R insult; the dark grey bar represented treatment with PRP before reperfusion; the light grey bar represented treatment with PRP after reperfusion

HIF-1α plays a crucial role in ischemic conditions and can promote angiogenesis by up-regulating VEGF expression. Our results showed that PRP significantly increased the tissue expression of HIF-1α in I/R-injured skin flaps compared to the corresponding PBS control, except in the post-PRP treatment groups (Fig. 3C–H). Notably, PRP pretreatment had the greatest effect on the induction of HIF-1α expression compared to PRP mid-treatment or post-treatment (Fig. 3D, F, H). PRP also significantly up-regulated HIF-1α expression in non-I/R groups (Fig. 3A, B). Moreover, PRP significantly elevated VEGF content compared to the corresponding PBS control from as early as day 1 to day 7, both with and without I/R insult, with the highest effect in the PRP pretreatment I/R group compared to PRP mid-treatment or post-treatment (Fig. 3I), indicating enhanced angiogenesis.

### PRP suppresses oxidative stress in I/R-injured skin flaps with the best response when administrated prior to I/R

**Fig. 4:**
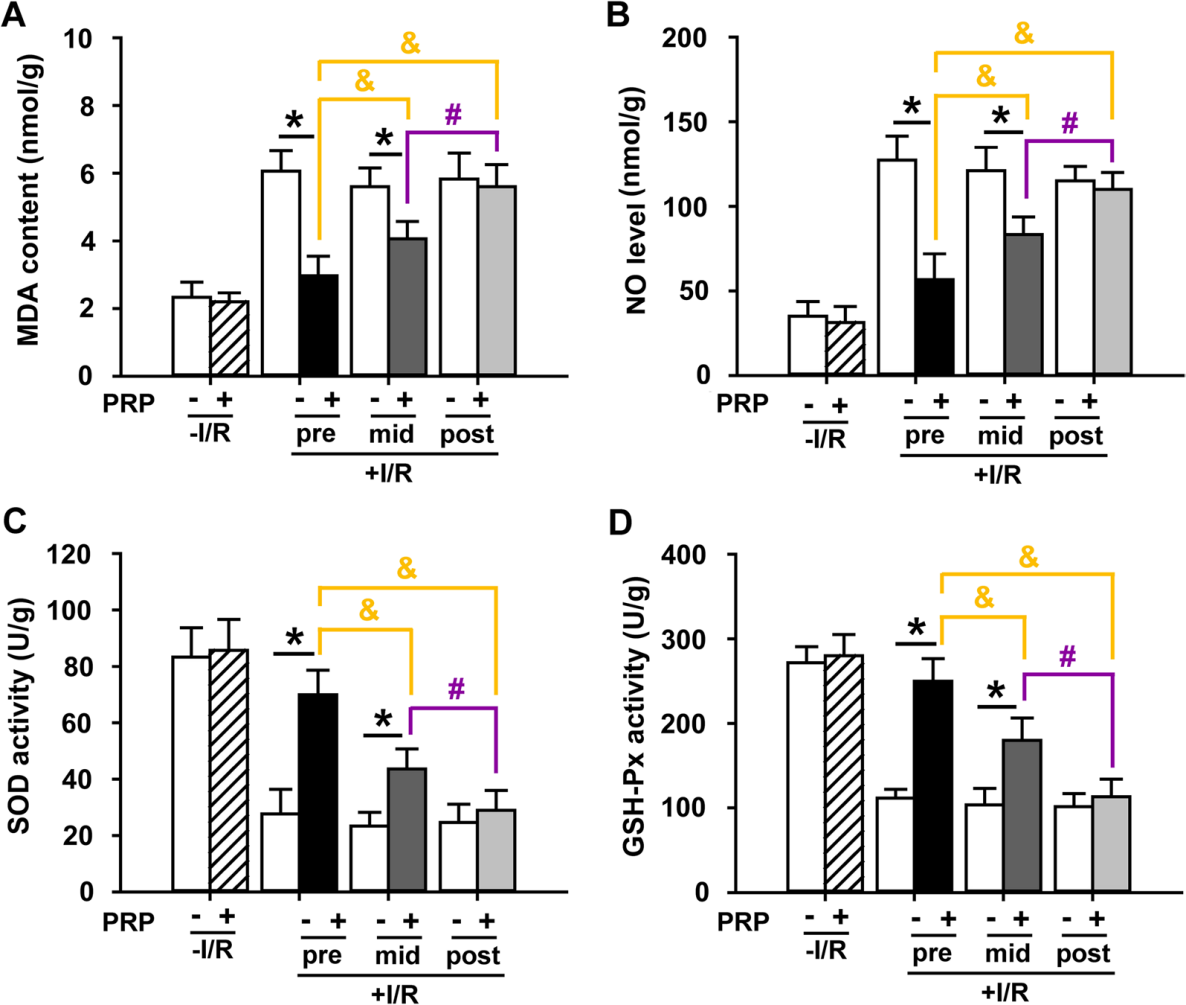
Evaluation of oxidative stress in I/R-injured skin flaps after PRP treatment. Tissue samples were obtained from each treatment group, weighed, homogenized, and subjected to biochemical assays for (**A**) MDA content, (**B**) NO level, (**C**) SOD activity, and (**D**) GSH-Px activity. Results were presented as nanomole per gram fresh weight, micromole per gram fresh weight, or unit per gram fresh weight. Error bars represent the means ± SD (n = 4). Student’s *t*-test for comparison between two groups, and one-way ANOVA followed by the Tukey–Kramer post hoc test for comparison among multiple groups were used. *p < 0.05 vs. the corresponding PBS control, ^&^*p* < 0.05 vs. the value in pre-PRP group after I/R insult, ^#^*p* < 0.05 vs. the value in mid-PRP group after I/R insult. In each bargraph, the white bars from left to right represented treatment with PBS without I/R insult, before I/R insult, before reperfusion, or after reperfusion, respectively; the slash bar represented treatment with PRP without I/R insult; the black bar represented treatment with PRP before I/R insult; the dark grey bar represented treatment with PRP before reperfusion; the light grey bar represented treatment with PRP after reperfusion

Several oxidative stress-related molecules that are widely recognized were measured to investigate the effect of PRP on oxidant injury. It was found that PRP significantly decreased the MDA content (Fig. 4A) and NO level (Fig. 4B) in I/R-injured flaps compared to the corresponding PBS control, except in the post-PRP treatment groups. In particular, PRP pretreatment showed the highest antioxidant effect compared to PRP mid-treatment or post-treatment (Fig. 4A, B). At the same time, PRP significantly enhanced the activities of SOD (Fig. 4C) and GSH-Px (Fig. 4D) in I/R-injured flaps compared to the corresponding PBS control, with the strongest effect in the PRP pretreatment group (Fig. 4C, D). It was worth noting that PRP did not affect these oxidative stress-related molecules in non-I/R groups (Fig. 4A–D).

### PRP suppresses inflammatory reaction in I/R-injured skin flaps with the best response when administrated prior to I/R

**Fig. 5:**
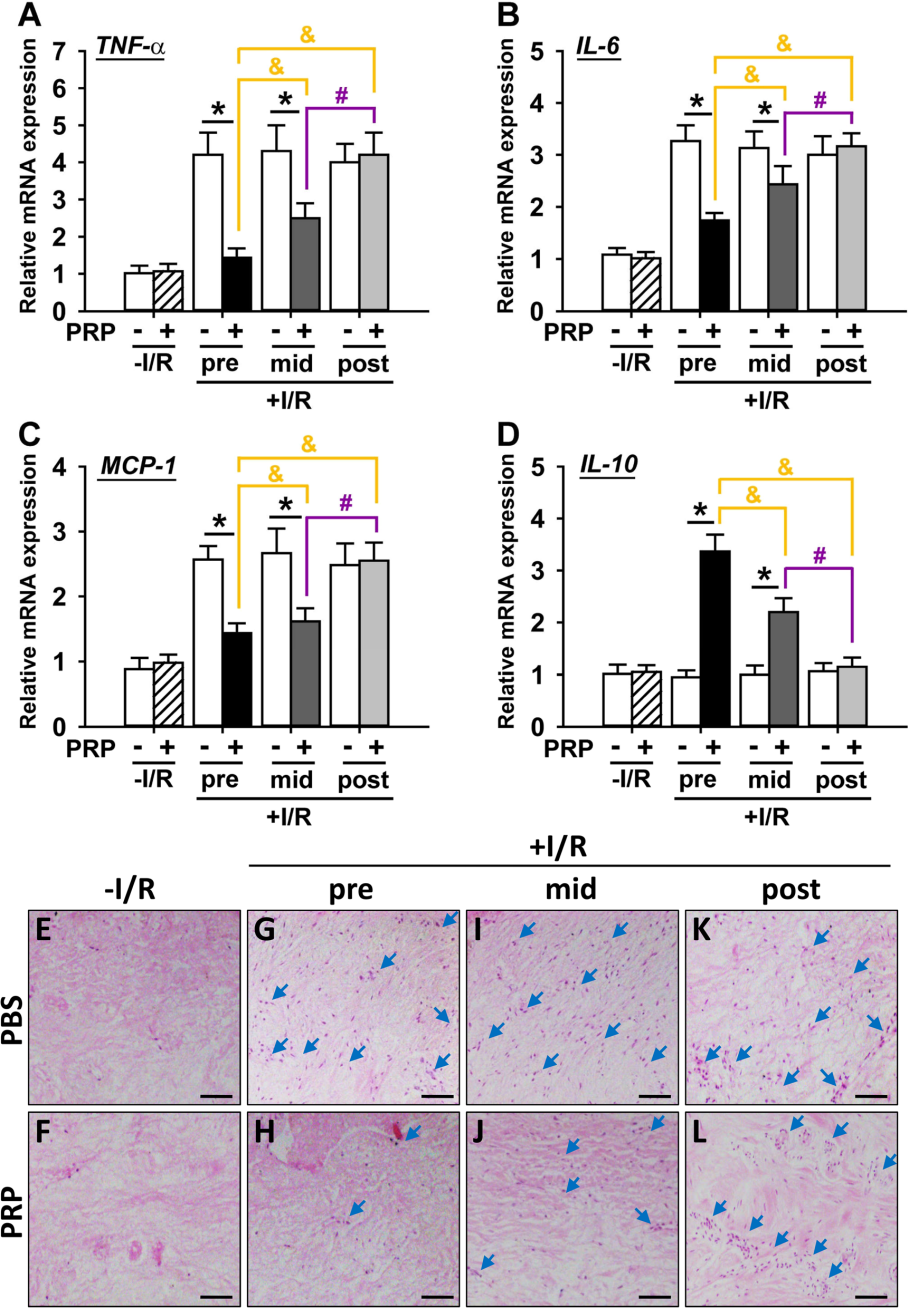
Evaluation of inflammatory responses in I/R-injured skin flaps after PRP treatment. The mRNA levels of pro-inflammatory cytokines, including (**A**) TNF-α, (**B**) IL-6, (**C**) MCP-1, as well as the anti-inflammatory cytokine (**D**) IL-10 were measured by qRT-PCR. Error bars represent the means ± SD (n = 4). Student’s *t*-test for comparison between two groups, and one-way ANOVA followed by the Tukey–Kramer post hoc test for comparison among multiple groups were used. *p < 0.05 vs. the corresponding PBS control, ^&^*p* < 0.05 vs. the value in pre-PRP group after I/R insult, ^#^p < 0.05 vs. the value in mid-PRP group after I/R insult. (**E–L**) The inflammatory infiltration (indicated by blue arrows) in different treatment groups was observed by H&E. Scar bar = 20 μm. In each bargraph, the white bars from left to right represented treatment with PBS without I/R insult, before I/R insult, before reperfusion, or after reperfusion, respectively; the slash bar represented treatment with PRP without I/R insult; the black bar represented treatment with PRP before I/R insult; the dark grey bar represented treatment with PRP before reperfusion; the light grey bar represented treatment with PRP after reperfusion

Several well-recognized inflammation-related cytokines were measured to investigate the effect of PRP on cellular inflammation. The results showed that PRP significantly inhibited the mRNA expression of pro-inflammatory tumor necrosis factor (TNF)-α (Fig. 5A), interleukin (IL)-6 (Fig. 5B), and monocyte chemotactic protein (MCP)-1 (Fig. 5C), while increased the mRNA expression of anti-inflammatory cytokine IL-10 (Fig. 5D) in I/R-injured flaps compared to the corresponding PBS control, except in the post-PRP treatment groups. Notably, PRP pretreatment had the strongest anti-inflammatory effect compared to PRP mid-treatment or post-treatment (Fig. 5A–D). Additionally, PRP did not affect these cytokines in non-I/R groups (Fig. 5A–D). H&E staining was further performed to verify the above results. It was shown that PRP substantially decreased the inflammatory cell infiltration of I/R-injured flaps compared to the corresponding PBS control, except in the post-PRP treatment groups (Fig. 5G–L), with the mildest inflammatory infiltration in the PRP pretreatment group compared to PRP mid-treatment or post-treatment (Fig. 5H, J, L). It was worth noting that little inflammatory infiltration was observed in both PRP and PBS-treated non-I/R groups (Fig. 5E, F).

### PRP inhibits the JAK/STAT signalling pathway in I/R-injured skin flaps with the best effect when administrated prior to I/R

**Fig. 6:**
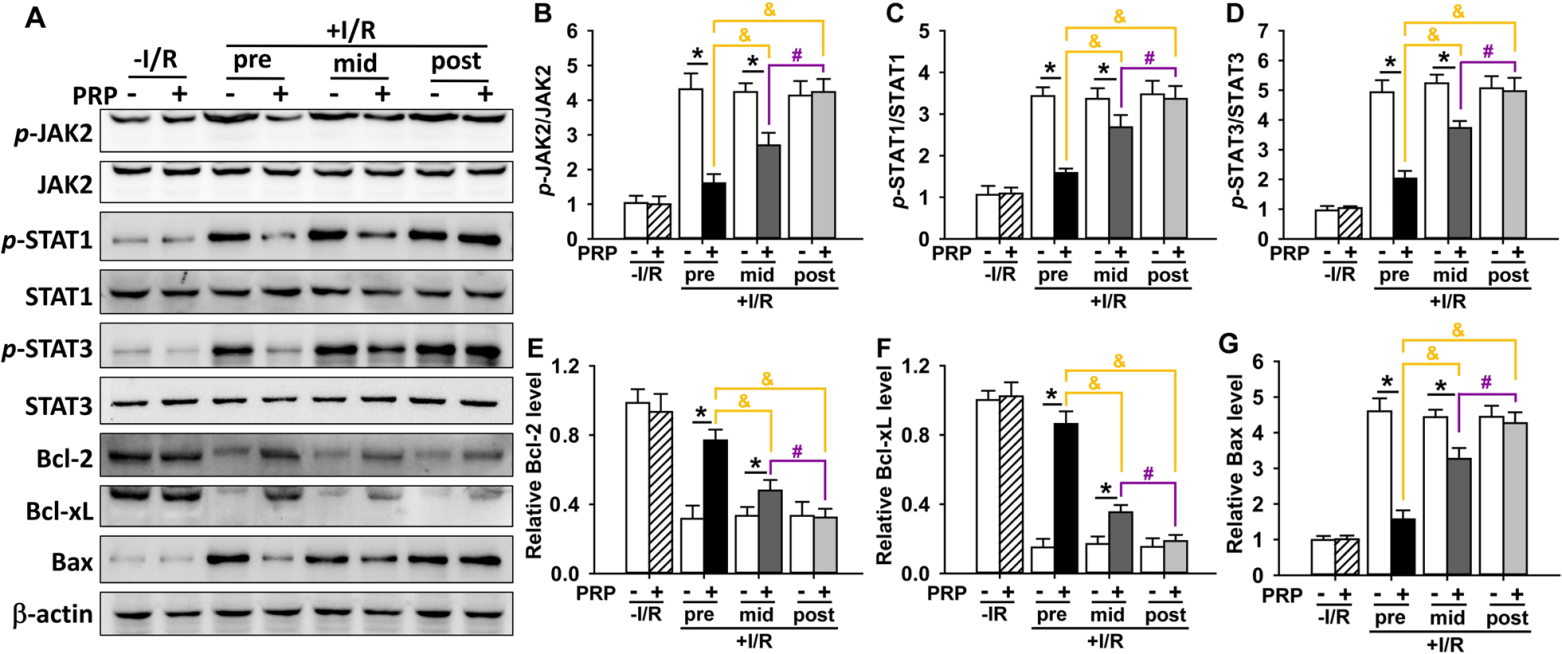
Evaluating the expression of proteins involved in the JAK/STAT pathway and apoptosis in I/R-injured skin flaps after PRP treatment. **A** Protein expression was measured by Western blotting using skin flap samples after PRP treatment in I/R-injured mice. β-actin served as an internal equal loading control. The ratios of **B** *p*-JAK2/JAK2, **C** *p*-STAT1/STAT1, and **D** *p*-STAT3/STAT3 were generated to determine the activation of JAK/STAT pathway. The relative protein levels of anti-apoptotic **E** Bcl-2 and **F** Bcl-xL, as well as pro-apoptotic **G** Bax were also quantified and present in bar graphs. Error bars represent the means ± SD (n = 4). Student’s *t*-test for comparison between two groups, and one-way ANOVA followed by the Tukey–Kramer post hoc test for comparison among multiple groups were used. *p < 0.05 vs. the corresponding PBS control, ^&^*p* < 0.05 vs. the value in pre-PRP group after I/R insult, ^#^*p* < 0.05 vs. the value in mid-PRP group after I/R insult. In each bargraph, the white bars from left to right represented treatment with PBS without I/R insult, before I/R insult, before reperfusion, or after reperfusion, respectively; the slash bar represented treatment with PRP without I/R insult; the black bar represented treatment with PRP before I/R insult; the dark grey bar represented treatment with PRP before reperfusion; the light grey bar represented treatment with PRP after reperfusion

The ratios of phosphorylated JAK/total JAK and phosphorylated STAT/total STAT were used to determine the activation of JAK and STAT, respectively (Arab et al. 2022). Here, PRP was found to significantly decrease the ratios of phosphorylated-JAK2 (*p*-JAK2)/JAK2 (Fig. 6A, B), phosphorylated-STAT1 (*p*-STAT1)/STAT1 (Fig. 6A, C) and phosphorylated-STAT3 (*p*-STAT3)/STAT3 (Fig. 6A, D) in I/R-injured flaps compared to the corresponding PBS control, except in the post-PRP treatment groups (Fig. 6A, B), indicating an inactivation of JAK/STAT signalling. In particular, PRP pretreatment showed the strongest inhibitory effect on JAK/STAT signalling compared to PRP mid-treatment or post-treatment (Fig. 6A, B). However, PRP did not affect the activation of JAK/STAT signalling in non-I/R groups (Fig. 6A, B).

### PRP alleviates cellular apoptosis in I/R-injured skin flaps with the best effect when administrated prior to I/R

**Fig. 7:**
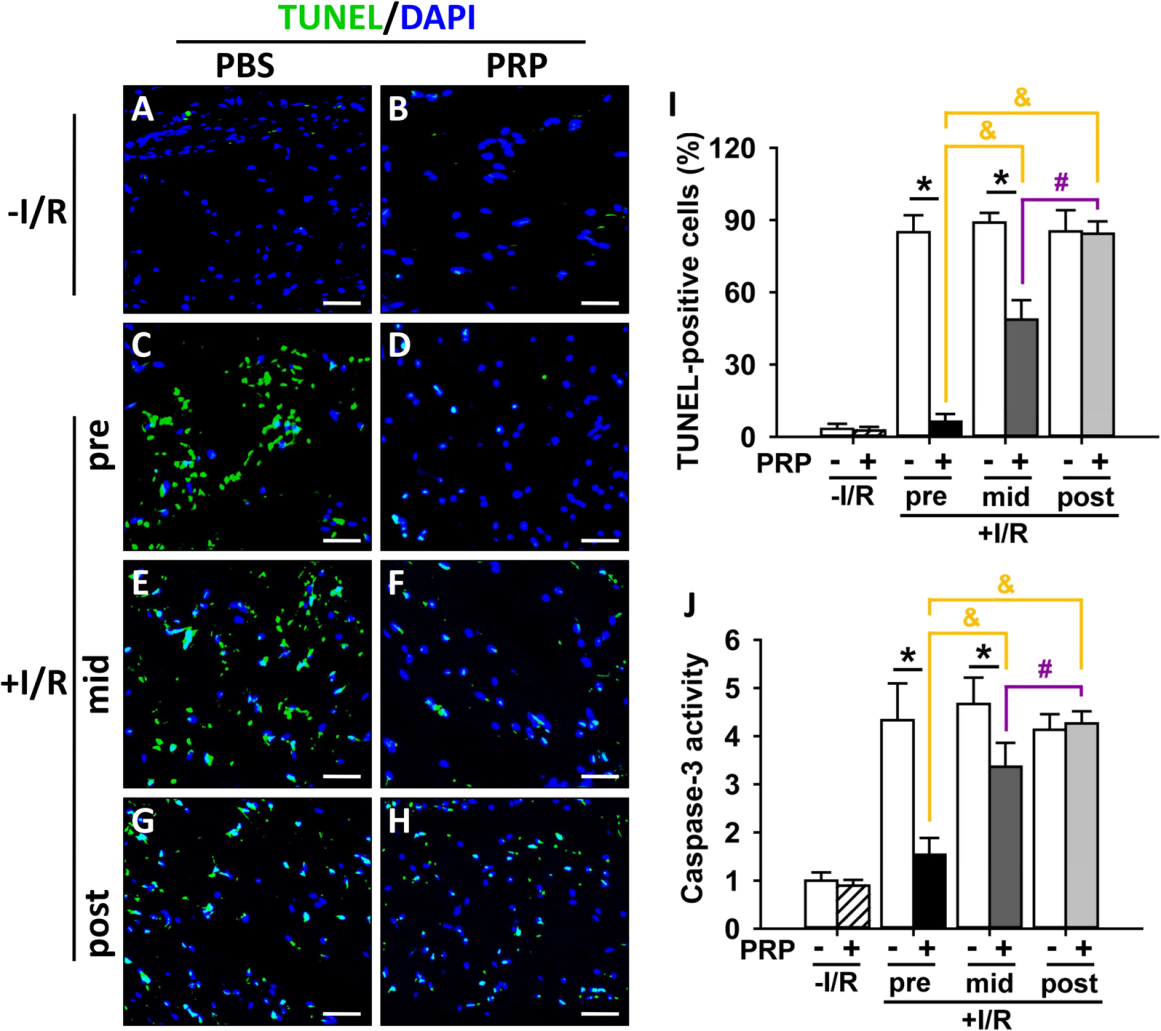
Assessment of apoptosis after PRP treatment in I/R-injured skin flaps. **A**–**H** The cellular apoptosis in skin flaps in different treatment groups was evaluated by TUNEL assay. The apoptotic cells were stained with *green* fluorescence. Nuclei were stained with DAPI (*blue*). Scar bar = 20 μm. **I** The percentage of TUNEL-positive cells in all treatment groups was calculated. **J** The activity of caspase-3 in all treatment groups was measured by a colorimetric assay kit. Error bars represent the means ± SD (n = 4). Student’s *t*-test for comparison between two groups, and one-way ANOVA followed by the Tukey–Kramer post hoc test for comparison among multiple groups were used. *p < 0.05 vs. the corresponding PBS control, ^&^*p* < 0.05 vs. the value in pre-PRP group after I/R insult, ^#^*p* < 0.05 vs. the value in mid-PRP group after I/R insult. In each bargraph, the white bars from left to right represented treatment with PBS without I/R insult, before I/R insult, before reperfusion, or after reperfusion, respectively; the slash bar represented treatment with PRP without I/R insult; the black bar represented treatment with PRP before I/R insult; the dark grey bar represented treatment with PRP before reperfusion; the light grey bar represented treatment with PRP after reperfusion

Several apoptotic markers were examined by Western blotting. It was shown that PRP substantially up-regulated the protein expression of anti-apoptotic B-cell lymphoma (Bcl)-2 (Fig. 6A, E) and Bcl-extra Larger (Bcl-xL) (Fig. 6A, F), while down-regulated the protein expression of pro-apoptotic Bcl-2-associated X protein (Bax) (Fig. 6A, G) in I/R-injured skin flaps compared to corresponding PBS control except in post-PRP treatment groups, with the most potent effect seen in PRP pre-treated group (Fig. 6A, E–G). These results were further substantiated by TUNEL assay. It was shown that PRP substantially reduced the number and percentage of TUNEL-positive cells in I/R-injured flaps compared to the corresponding PBS control except in post-PRP treatment groups (Fig. 7A–I), with the sharpest reduction in PRP pre-treated group (Fig. 7D, F, H, I). Moreover, the activity of caspase-3 in I/R-injured flaps was also suppressed by pre-PRP and mid-PRP treatment but not by post-PRP treatment, with the most significant suppression in PRP pretreatment group (Fig. 7J), indicating an alleviation of the cellular apoptosis. It is worth to note that PRP did not affect above apoptosis-related factors in non-I/R groups (Figs. 6, 7).

### PRP pretreatment obtains better efficacy on deactivating JAK/STAT signalling and alleviating apoptosis than AG490 in I/R-injured flaps

**Fig. 8:**
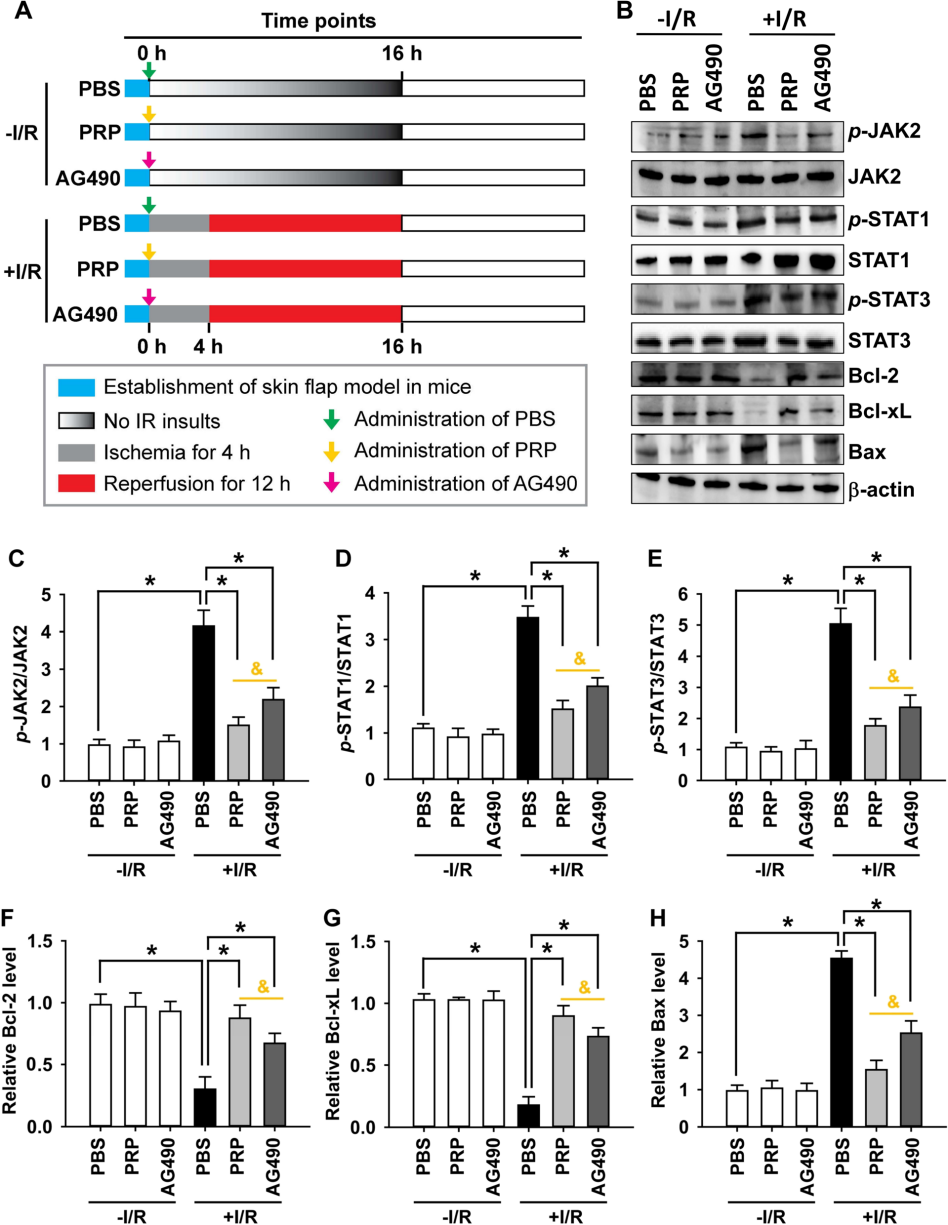
Comparison of PRP pre-treatment with AG490, a putative JAK/STAT inhibitor, in deactivating JAK/STAT signalling and alleviating apoptosis in I/R-injured skin flaps. **A** Experimental protocol: twenty-four mice were randomly and evenly divided into six groups as described in “Materials and methods” section. PBS (green arrows), PRP (yellow arrows), or AG490 (red arrows) was subcutaneously injected to the flaps prior to I/R insult or without I/R insult. Skin flap samples were collected and processed at 16 h on day 0 for Western blotting. Blue rectangles represent the period for model establishment. Gray rectangles represent the period of 4-h ischemia. Red rectangles represent the period of 12-h reperfusion. Gradient gray rectangles represent no I/R insult. **B** Protein expression in six treatment groups was assessed by Western blotting. β-actin served as an internal equal loading control. The ratios of **C** *p*-JAK2/JAK2, **D** *p*-STAT1/STAT1, and **E** *p*-STAT3/STAT3 were calculated to determine the activation of JAK/STAT pathway. The relative protein levels of **F** Bcl-2, **G** Bcl-xL, and **H** Bax were also quantified and present in bar graphs. Error bars represent the means ± SD (n = 4). Student’s *t*-test for comparison between two groups, and one-way ANOVA followed by the Tukey–Kramer post hoc test for comparison among multiple groups were used. *p < 0.05 vs. the value in PBS group after I/R insult, ^&^*p* < 0.05 vs. the value in PRP pre-treatment group after I/R insult. In each bargraph, the white bars from left to right represented pretreatment with PBS, PRP or AG490 without I/R insult, respectively; the black bar represented pretreatment with PBS with I/R insult; the light grey bar represented pretreatment with PRP with I/R insult; the dark grey bar represented pretreatment with AG490 with I/R insult

Based on the above findings, it was shown that PRP administrated prior to I/R had overall superior benefits compared to mid-PRP or post-PRP treatment in improving flap survival and blood perfusion, repressing oxidative stress and inflammation, as well as alleviating apoptosis and deactivating JAK/STAT. Therefore, here we only compared the efficacy of PRP pretreatment with AG490, a potent and specific inhibitor of JAK/STAT. A skin flap model was established in mice, followed by either no I/R or with I/R insults (Fig. 8A). The results showed that I/R significantly promoted the phosphorylation of JAK2, STAT1 and STAT3, as well as increased the ratios of *p*-JAK2/JAK2, *p*-STAT1/STAT1 and *p*-STAT3/STAT3. Importantly, PRP pretreatment was significantly more effective than AG490 in reversing these phosphorylation events (Fig. 8B–E). Additionally, I/R significantly reduced the protein expression of anti-apoptotic Bcl-2 and Bcl-xL, while increased the expression of pro-apoptotic Bax. Importantly, PRP showed a stronger ability than AG490 to reverse these changes in protein expression (Fig. 8B, F–H).

## Discussion

I/R injury commonly occurs during microsurgical free flap transfer, organ transplantation, and other major surgeries, causing organ compromise or even permanent dysfunction (Fernández et al. 2020). Therefore, it is crucial to reveal the underlying mechanisms and seek effective interventions to prevent or attenuate I/R-induced injury. This study evaluated the role of PRP in a mouse dorsal pedicled flap model subjected to I/R insult. The results showed that PRP treatment significantly increased the survival area of the flap compared to PBS control when administrated before I/R or before reperfusion on day 4 and 7 post-surgery, but no differences were observed when administrated during reperfusion phase, suggesting that PRP exerts functions mainly at the early I/R stages. PRP treatment also promoted flap survival even the flap did not experience I/R insult on day 4 and 7 post-surgery, indicating the favorable role of PRP in the stimulation derived from model establishment. PRP treatment significantly increased blood perfusion in the flap compared to PBS control except in PRP post-treatment groups, this phenomenon was observed as early as on day 1 in PRP pretreatment groups, indicating that PRP-mediated protection is more likely to be preventive rather than curative when ischemia or I/R already initiates. Although prolonged ischemia may improve angiogenesis by opening choke vessels, reperfusion can exacerbate tissue damage and necrosis (Cohen et al. 2016). Therefore, minimizing reperfusion-induced injury is crucial for flap surgery and organ transplantation to preserve tissue integrity.

Oxidative stress plays a key role in I/R-induced tissue injury (Orellana-Urzúa et al. 2020). Reactive oxygen (ROS) and nitrogen (RNS) species produced in large amounts can enhance lipid peroxidation, damage cell and mitochondria membranes, and induce keratinocyte and fibroblast apoptosis (Liu et al. 2022). Therefore, reducing the damage caused by I/R-induced oxidative stress may effectively alleviate the occurrence of chronic ischemic wounds. Studies have shown that I/R can induce excessive production of MDA, NO, and ROS, while reduce the expression of endogenous antioxidant enzymes such as SOD and GSH-Px, resulting in oxidative stress in tissues (Sun et al. 2015). SOD catalyzes the incorporation of O^2−^ into H_2_O_2_ and GSH-Px is a potent ROS scavenger. The enhanced activities of these endogenous antioxidant enzymes provide protection against oxidative stress. In this study, I/R dramatically up-regulated MDA and NO levels while down-regulated SOD and GSH-Px activities, indicating redox imbalance and high levels of ROS occur in I/R-injured flaps. PRP administrated prior to I/R or before reperfusion reversed the increase in levels of MDA and NO, and reversed the decrease in activities of SOD and GSH-Px. PRP administrated during the reperfusion did not differ from the PBS control. These results suggest that PRP pretreatment could enhance the endogenous antioxidant capacity and suppress oxidative stress in the flap after I/R insult. It is also worth noting that PRP treatment did not affect the levels of MDA and NO, as well as the activities of SOD and GSH-Px in the non-I/R groups, indicating that PRP’s protective role might be stimuli/injury-responsive.

Inflammatory cells produce various pro-inflammatory mediators, including MCP-1, IL-1β, and IL-6, and infiltrate tissues during the early phase of the response to I/R. α-Tocopherol-mediated preservation of cardiac function against I/R injury involves decreased neutrophil infiltration and a systemic anti-inflammatory shift to Ly6C^low^ monocytes (Wallert et al. 2019). In this study, I/R injury up-regulated the mRNA expression of pro-inflammatory cytokines TNF-α, IL-6, and MCP-1, but did not affect the mRNA level of the anti-inflammatory cytokine IL-10, indicating exacerbated inflammatory responses upon I/R insult. PRP administrated prior to I/R or before reperfusion attenuated the increase of pro-inflammatory cytokines and effectively promoted the mRNA level of IL-10. PRP treatment during the reperfusion phase did not differ from PBS control. These results suggest that PRP pretreatment can suppress inflammation in the flap after I/R insult by decreasing the release of inflammatory mediators. PRP treatment did not affect the mRNA expression of inflammatory mediators in the non-I/R groups, indicating that I/R injury initiates inflammatory responses. In agreement with our results, Allam et al. reported that PRP can preserve ovarian function and structure against I/R-induced ovarian injury by suppressing TNF-α-mediated inflammation (Allam et al. 2022). Neutrophil recruitment and infiltration are aggravated during the early phase of the response to I/R injury, but PRP treatment significantly reduces this phenomenon, resulting in an improvement in I/R-induced pathology. Rah et al. also observed that PRP treatment abolishes ischemia-induced neutrophil accumulation and aggregation, extensive hyperemia, and microthrombus formation (Rah et al. 2017).

The JAK/STAT pathway is a stress-responsive mechanism that transduces extracellular signals to the nucleus in response to growth factors and cytokines (Bolli et al. 2003). Its activation is a significant contributor to I/R-induced injury, and interference with its activation enhances tissue function recovery. Edaravone (Zhao et al. 2020), propofol (Chen et al. 2019) and tetramethylpyrazine (Gong et al. 2019) have been shown to protect against I/R injury by inhibiting the JAK/STAT pathway. In this study, I/R significantly activated JAK/STAT signaling, but PRP administrated prior to I/R or before reperfusion reversed this effect by inhibiting phosphorylation. PRP administrated during reperfusion had little effect on JAK/STAT activation. AG490, a selective JAK inhibitor, further confirmed the role of PRP in regulating JAK/STAT signaling, and PRP was more effective than AG490 in abolishing JAK/STAT phosphorylation, providing a novel therapeutic strategy for the treatment of JAK/STAT-medicated skin flap I/R injury.

Apoptosis is another key event in I/R injury, with ROS activating apoptosis cascades during reperfusion (Orellana-Urzúa et al. 2020). For example, asiatic acid protects against myocardial I/R injury via mitochondrial apoptosis pathway (Yi et al. 2022). ROS activate mitochondrial-mediated neuronal apoptosis and disrupt the blood–brain barrier in cerebral I/R injury (He et al. 2020). In this study, I/R activated caspase-3, suppressed anti-apoptotic Bcl-2 and Bcl-xL, and enhanced pro-apoptotic Bax, leading to apoptois. Subcutaneous PRP administration attenuated apoptotic cell death when treated during early I/R. Samy et al. also reported PRP exerting cytoprotective and therapeutic roles against torsion/detorsion-induced testicular I/R injury through apoptosis reduction (Samy et al. 2020). It is worth noting that PRP’s anti-apoptotic effect is even more significant than AG490, suggesting a second pathway involved in PRP-mediated protection against I/R injury other than JAK/STAT.

Several medications or dietary supplements have been proposed to improve I/R-injured tissue functions. ATP-MgCl_2_ restores energy-rich phosphates and improves mitochondrial energy metabolism and liver function after ischemia (Jeong and Lee 2000). Lomodex-MgSO_4_ protects against testicular torsion-induced injury and improves semen quality, fertility and long-term health (Adivarekar et al. 2005). Antioxidant dietary supplements, such as multi-vitamins, green tea, fruits, and vegetables, are suggested to enhance endogenous oxidative defenses against ROS-mediated I/R injury (Valaei et al. 2021). Dietary nitrate from green vegetables alleviates inflammation-mediated I/R injury by attenuating macrophages and neutrophils and decreasing pro-inflammatory cytokines (Cui et al. 2020). However, these procedures are expensive, complex, require clinical trials, or have limited efficacy. In contrast, PRP is inexpensive, easy to obtain and handle, with no risk of host rejection or immune reactions when autologous (Etulain 2018; Everts et al. 2020). Furthermore, PRP has an antimicrobial action with low risk of infection (Sethi et al. 2021). PRP is now used in various clinical situations, including periodontal osseous defects (Reshma et al. 2022), maxillofacial reconstruction (Farshidfar et al. 2022), spinal fusion (Kawabata et al. 2023), cardiovascular surgery (Bai et al. 2020), and chronic ulcer treatment (He et al. 2022).

### Limitations of the study

This study has several limitations. Firstly, homologous allogenic PRP from other mice instead of autologous one was used, which could elicit an immune response and affect the results (Liu et al. 2002). However, previous studies have shown that homologous PRP still provides positive benefits despite this potential drawback (Yamaguchi et al. 2012). Secondly, the small size of laboratory mice prevented serial measurements of serum components, which would have allowed us to analyze the systemic effect of PRP on the whole body over time without causing anemia. Thirdly, mice were euthanized on day 7 post-I/R, preventing long-term survival studies or analysis of the long-term effects of PRP on skin flap survival. Finally, PRP studies lack standardization, with differences in source, platelet and white blood cell count, and growth factor level, affecting PRP activity. There is also no consensus on the stability of PRP during storage, the necessity of an activating agent, or the optimal method and location of administration. Therefore, additional studies are needed to justify the use of PRP without bias.

## Conclusions

In this study, we used a mouse axial flap model to investigate the effect of PRP on I/R-injured skin flaps. We found that PRP protected against flap injury by improving survival, blood perfusion and angiogenesis, reducing oxidative stress and inflammation, and attenuating apoptosis, which was mediated at least partly via deactivating JAK/STAT signaling pathway. PRP administration prior to I/R was optimal for exerting its protective effect. PRP could represent a novel and effective therapy for managing I/R-induced flap injury, highlighting its potential value for clinical application.

## Data Availability

The data that support the findings of this study are available on request from the corresponding author upon reasonable request.
